# Electromagnetic Initiation and Propagation of Bipolar Radiofrequency Tissue Reactions via Invasive Non-Insulated Microneedle Electrodes

**DOI:** 10.1038/srep16735

**Published:** 2015-11-13

**Authors:** Jongju Na, Zhenlong Zheng, Christopher Dannaker, Sang Eun Lee, Jin-Soo Kang, Sung Bin Cho

**Affiliations:** 1 Department of Anatomy, Soonchunhyang University College of Medicine, Cheonan, Korea; 2 Department of Dermatology and Cutaneous Biology Research Institute, Yonsei University College of Medicine, Seoul, Korea; 3 Department of Dermatology, Yanbian University Hospital, Yanji, China; 4 Department of Dermatology, University of California, School of Medicine, San Francisco, CA, USA; 5 Kangskin Dermatology Clinic, Seoul, Korea

## Abstract

Radiofrequency (RF) energy can be emitted into the skin, either non- or invasively, via a monopolar mode that utilizes an active electrode and a grounded electrode or via a bipolar mode that employs two active electrodes. In this experimental study of RF tissue reactions, bipolar RF energy was emitted *in vivo* to micropig skin at varying microneedle penetration depths, signal amplitudes, and conduction times. Immediately after RF treatment, skin samples exhibited RF-induced coagulation columns of thermal injury, separately generated around each microneedle in the dermis. In *ex vivo* bovine liver tissue, the thermal coagulation columns were found to be concentrated maximally around the pointed tips of each electrode. After a RF conduction time of 2 seconds, the individual areas of thermal coagulation began to converge with neighboring RF-induced coagulation columns; the convergence of coagulation columns was found to start from the tips of neighboring electrodes.

Radiofrequency (RF) refers to high frequency alternating electrical current at the frequency range traditionally used for radio-wave communication. Electromagnetic signal, including RF, induces an electrothermal reaction in targeted tissues, the patterns of which depend on the resistance of the tissue^1^. RF energy can be emitted into the skin either non- or invasively via a monopolar mode that utilizes an active electrode and a grounded electrode or via a bipolar mode that employs two active electrodes^2^. In monopolar modes, an electrical circuit formed by an electron current that flows from the active electrode to the grounded electrode is generated in the patient’s body^2^. Meanwhile, bipolar electrosurgery systems induce an electrical circuit between the two active electrodes that is limited to regionally targeted tissues^2^,^3^. In the skin, these active electrodes emit an electron current that flows through the shortest path in the target tissue between the electrodes, theoretically limiting the depth of the thermal response induced by the electromagnetic energy^2^.

Invasive RF systems, which deliver electromagnetic energy through electrodes that penetrate into target tissues, offer advantages of a deeper layer of treatment in a non-contiguous pattern, compared with noninvasive systems^4^,^5^,^6^,^7^. In a recent study, a monopolar 0.4-MHz RF system equipped with a single penetrating electrode induced thermal coagulation in *ex vivo* bovine liver tissue that started from the tips of non-insulated electrodes and formed a rim of coagulated tissue around the entire length of the needles with increasing energy levels^6^. Therein, the thickest rim of coagulated tissue was formed around the tips of the electrodes, suggesting that non-insulated penetrating electrodes can be used to effectively and safely deliver RF energy to the skin while preserving the epidermis^6^.

Meanwhile, research on invasive bipolar RF devices has suggested that electrodes must be insulated in order to preserve the epidermis^5^,^6^. In a predictive model, a minimally invasive bipolar RF system showed a thermal profile that was confined between the non-insulated portions of the penetrating electrodes^5^. Moreover, in an *in vivo* micropig study, the emission of RF energy via a bipolar microneedle RF device equipped with insulated penetrating electrodes induced individual coagulation columns, a water drop-shaped oval zone of thermal injury, at the tip of each electrode in the dermis immediately after treatment^7^. However, further investigation into why the coagulation columns were generated separately around each electrode instead of between electrodes was not conducted^7^.

In this experimental study of RF tissue reactions, we aimed to investigate the electromagnetic patterns of initiation and propagation of bipolar RF energy delivered through invasive non-insulated microneedle electrodes. Bipolar RF energy was emitted to the *in vivo* skin of a micropig at varying microneedle penetration depths, RF signal amplitudes, and RF conduction times. Additionally, *ex vivo* bovine liver tissue study was performed to investigate histological changes in the liver tissue induced by the passing of electrical current between microneedle electrodes. Meanwhile, high-speed cinematographs were captured to demonstrate time-dependent tissue reactions of RF energy on *ex vivo* micropig muscle and bovine liver tissues.

## Results

### *In vivo* tissue reactions in micropig skin

**Figure 1:**
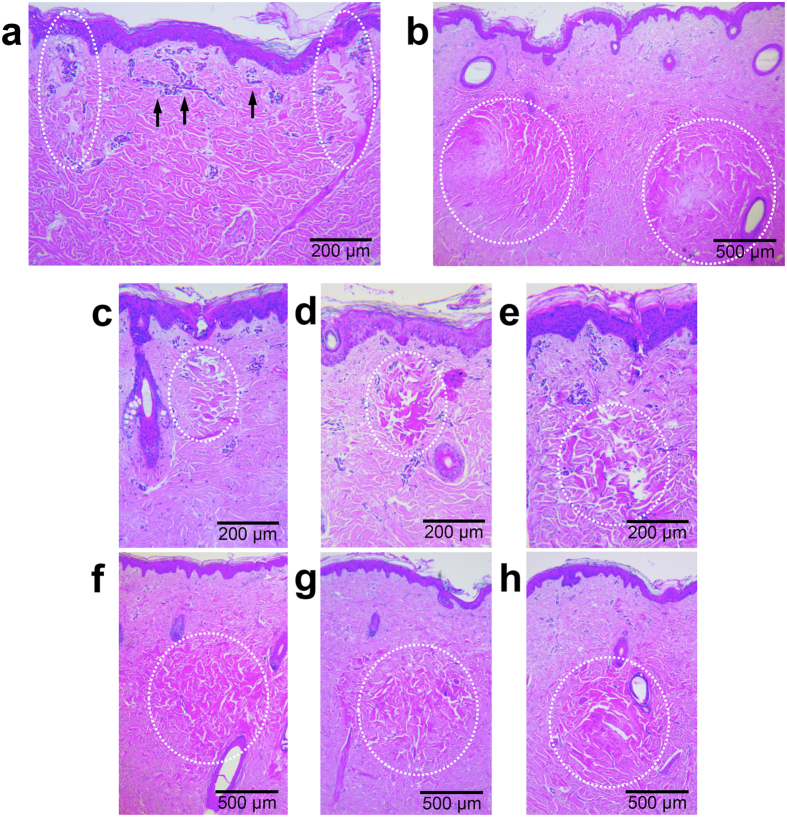
Tissue reactions after invasive bipolar radiofrequency (RF) treatment using non-insulated microneedle electrodes on *in vivo* micropig skin. RF-induced coagulation columns (broken lines) of thermal injury are generated separately around each microneedle electrode (**a**) in the upper dermis with noticeable vascular congestion between the electrodes (arrows) and (**b**) in the mid dermis with the treatment settings of a 3.0-mm penetration depth, a signal amplitude of 29.6 V, and a conduction time of 300 msec. Photomicrographs of micropig skin sections for a RF conduction time of (**c**) 120 msec, (**d**) 200 msec, and (**e**) 300 msec at a signal amplitude of 35.6 V and a electrode penetration depth of 1.0 mm. Photomicrographs of micropig skin sections for a signal amplitude of (**f**) 29.5 V, (**g**) 32.6 V, or (**h**) 35.6 V, with a conduction time of 200 msec and an electrode penetration depth of 2.0 mm. H&E stain.

Immediately after RF treatment, skin samples exhibited coagulation columns of thermal injury, generated separately around each microneedle electrode in the dermis (Fig. 1a,b). No noticeable RF-induced tissue reactions were observed in the epidermis or between the electrodes in the dermis. Photomicrographs of skin sections stained with hematoxylin and eosin were analyzed according to RF signal amplitudes ranging from 25.6 V to 36.6 V and conduction times of 120 msec, 200 msec, and 300 msec. At the same penetration depth and RF signal amplitude, longer RF conduction times created larger areas of coagulation in the dermis (Fig. 1c–e). Meanwhile, higher RF energy generated greater degrees of tissue destruction at the same microneedle penetration depth and RF conduction time (Fig. 1f–h).

### *Ex vivo* tissue reactions in bovine liver tissue

**Figure 2:**
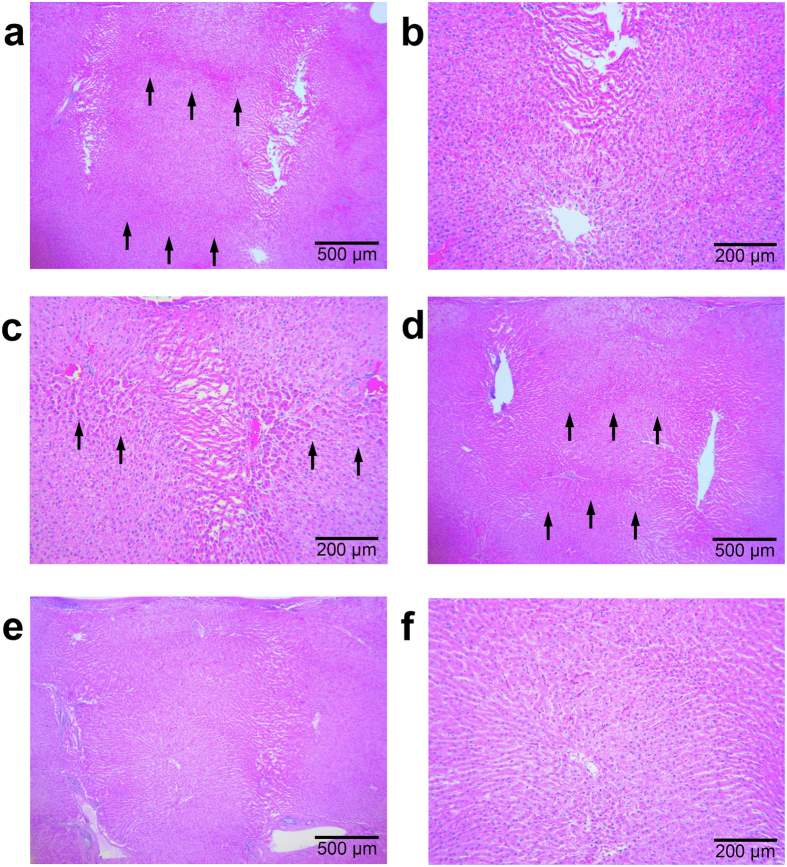
Tissue reactions after invasive bipolar RF treatment utilizing non-insulated microneedle electrodes on *ex vivo* bovine liver tissue. Individual areas of thermal coagulation converge on neighboring coagulation columns (arrows), (**a**) after 2 seconds of RF conduction at an electrode penetration depth of 3.0 mm and a signal amplitude of 31.6 V, (**b**) originating from the tips of neighboring microneedle electrodes. (**c**) Electrical currents start to converge bilaterally to neighboring electrodes after 2 seconds of RF conduction at an electrode penetration depth of 3.0 mm and a signal amplitude of 36.6 V. The paths of the electrical currents between coagulation areas become more apparent after (**d**) 3 and (**e**) 4 seconds of RF conduction at an electrode penetration depth of 3.0 mm and a signal amplitude of 31.6 V. (**f**) RF conduction for more than 4 seconds shows the propagation of electrical current through the entire length of the electrode with realignment of hepatocytes along the path of the current. H&E stain.

Tissue responses induced by treatment with bipolar RF signals emitted via invasive non-insulated microneedle electrodes over conduction times of 120 msec, 200 msec, 300 msec, and 1–7 seconds, in increments of 1 second, were investigated in *ex vivo* bovine liver tissue. Liver tissue is mainly composed of hepatocytes and vasculatures with relatively homogeneous tissue impedance and permittivity compared to skin tissue. However, oval and cocoon-shaped coagulation columns, which were generated around each microneedle electrode, were observed immediately after the emission of RF energy for 120 msec, 200 msec, and 300 msec, as seen in the *in vivo* micropig skin experiments (data not shown). At earlier conduction times, thermal coagulation columns were concentrated around the pointed tips of each individual electrode. Then, after 2 seconds of RF conduction time, individual areas of thermal coagulation converged with neighboring RF-induced coagulation columns (Fig. 2a–d). The converging of the coagulation columns was found to start from the pointed tips of the neighboring electrodes, and became apparent along the shortest path between the tips of the closest neighboring electrodes. An additional path of electrical current between coagulation areas was also found around the middle of the microneedle electrodes. At RF conduction times of longer than 4 seconds, circuits of electrical current were found throughout the entire length of the electrode, traversing adjoining coagulation areas, as seen in the realignment of hepatocytes (Fig. 2e,f). At the point in the RF treatment when individual coagulation areas began to converge with another, electrical currents showed propagation to all neighboring electrodes, not just between each pair of electrodes (Fig. 2c).

**Figure 3:**
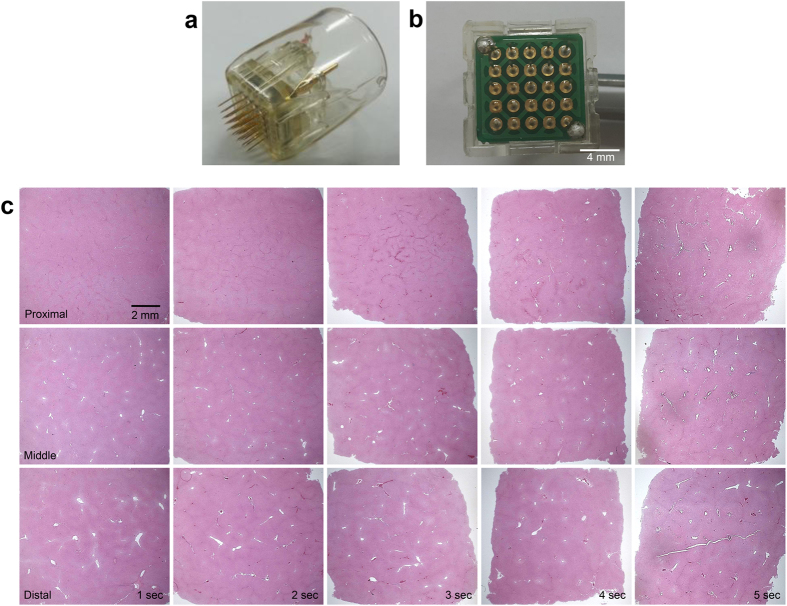
Horizontal sections of the *ex vivo* bovine liver tissue. (**a**) Disposable tips used by the bipolar RF device comprise 25 invasive non-insulated electrodes (**b**) arranged in a 5 × 5 pattern. (**c**) Tissue sections of bovine liver were obtained after RF treatment with a penetration depth of 3.0 mm and a signal amplitude of 36.6 V. Remarkable carbonization is observed along the distal ends of penetrating electrodes after 1 second of RF conduction. Along the middle of the electrodes, tissue coagulation is visible at the conduction time of 1 second, and remarkable carbonization seems to develop after 3 seconds. At the proximal ends of the electrodes, distinguishable congestion of vascular structures is observed on the specimens treated over RF conduction times of 2 and 3 seconds. Coagulation along superficial portions of the *ex vivo* bone liver tissue is found after RF treatment with a conduction time of 4 seconds, while remarkable carbonization appears after the conduction time of 5 seconds. H&E stain.

Horizontal sections of the *ex vivo* bovine liver tissue were obtained immediately after treatment with the RF settings of a penetration depth of 3.0 mm, a signal amplitude of 36.6 V, and a conduction time of 1–7 seconds in increments of 1 second (Fig. 3). Overall, noticeable shrinkage of the bovine liver tissue was found with increasing conduction time. Remarkable carbonization was observed at the RF conduction time of 1 second on the distal ends of the penetrating electrodes. Along the middle parts of the electrodes, tissue coagulation was noted in the RF-treated liver tissue at the conduction time of 1 second, while remarkable carbonization developed after 3 seconds. At the proximal ends of the electrodes, tissue coagulation along the superficial portion of the *ex vivo* bone liver tissue was discovered after RF treatment for 4 seconds; remarkable carbonization developed after 5 seconds.

### High-speed cinematography

High-speed cinematography study was performed to capture the effects of invasive bipolar RF treatment using non-insulated microneedle electrodes on *ex vivo* micropig muscle and bovine liver tissue, from the initial tissue reactions to the final reactions of convergence and vaporization, over continuous shooting frames. In both tissues, tissue reactions were initiated at the tips of the microneedle electrodes that penetrated into the tissues (Supplementary Video 1). Thereafter, tissue reactions propagated upward along the entire length of the microneedle electrodes in two phases: As the reactions propagated up from the tips of the microneedle electrodes, they expanded to deeper, wider, and higher areas around the microneedle. After a brief delay, the second propagation phase began as reactions continued to move upward around the body of the microneedle electrode and expand laterally. The propagation of inter-electrode currents between neighboring electrodes became apparent first between the tips of the electrodes, then second between the mid-portions of the electrodes, and lastly between the entire length of the electrodes. After complete convergence of all individual coagulation areas, tissues then began to show vaporization and carbonization.

### Effects on vascular structures and hair follicles

**Figure 4:**
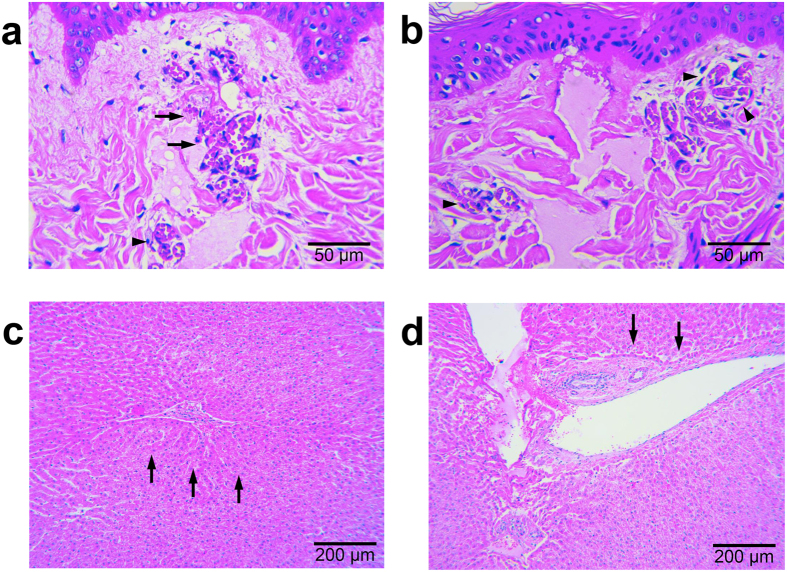
Effect of 2-MHz invasive bipolar RF treatment using non-insulated microneedle electrodes on vascular structures. Photomicrographs of micropig skin sections stained with hematoxylin and eosin after RF treatment at a 3.0-mm penetration depth, a signal amplitude of 29.6 V, and a conduction time of 300 msec demonstrate (**a**) partial extravasation of red blood cells around upper dermal vascular structures due to direct injury from the microneedle, as well as (**b**) remarkable congestion, but no destruction: vasculatures inside or near coagulation columns. (**c**) RF signals were preferentially conducted along penetrating vessels of *ex vivo* bovine liver tissue, treated with the settings of a 3.0-mm penetration depth, a signal amplitude of 31.6 V, and a conduction time of 3 seconds. (**d**) RF-induced coagulation is found mainly in the tunica adventitia, not in endothelial cells, the tunica intima, or the tunica media at a 3.0-mm penetration depth, a signal amplitude of 31.6 V, and a conduction time of 4 seconds. H&E stain.

Around upper dermal vascular structures, which were directly damaged by the insertion of the microneedle, partial extravasation of red blood cells was apparent (Fig. 4a); however, most of the vascular structures inside or near the coagulation columns were only congested, not destructed (Fig. 4b). Additionally, while marked vascular congestion was found in the upper dermis along regions directly between the electrodes (Fig. 1a), no distinguishable tissue coagulation was found in the tissue located between the electrodes surrounding the congested vascular structures. Moreover, RF signals were preferentially conducted along penetrating vessels in the bovine liver tissue, and RF-induced coagulation was found mainly in the tunica adventitia, not in endothelial cells, the tunica intima, or the tunica media (Fig. 4c,d). Noticeable congestion of smaller blood vessels was also found inside or near the coagulation columns around each electrode, as seen in the *in vivo* micropig skin study. Horizontal sections of the *ex vivo* bovine liver tissue exhibited no remarkable changes around the proximal ends of the penetrating electrodes at the RF conduction time of 1 second (Fig. 3). At the RF conduction times of 2 seconds and 3 seconds, distinguishable congestion of vascular structures was observed in the specimens, after which, at 4 seconds, remarkable tissue coagulation surrounding the entire electrode was discovered.

**Figure 5:**
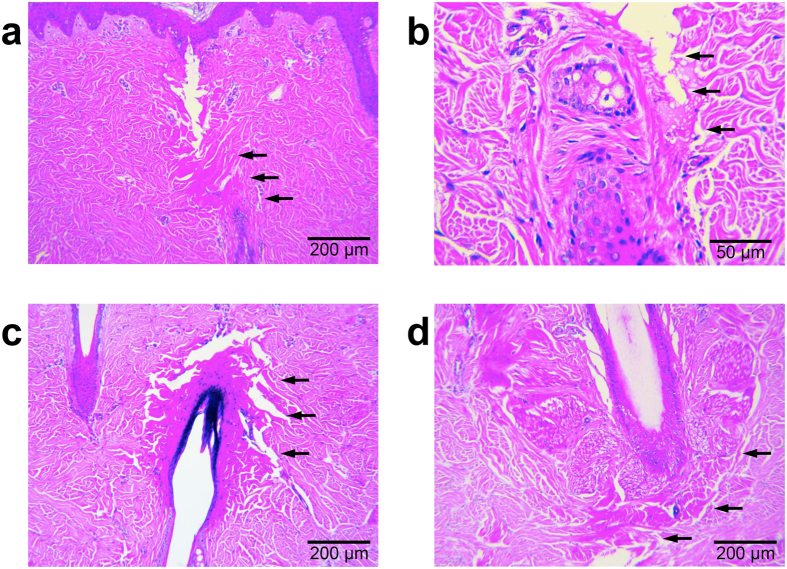
Effect of 2-MHz invasive bipolar RF treatment using non-insulated microneedle electrodes on hair follicles. Photomicrographs of micropig skin sections stained with hematoxylin and eosin after RF treatment show that RF signals tend to propagate along the outside of hair follicles, mainly the outer root sheath and fibrous connective tissue, (**a**) at a 3.0-mm penetration depth, a signal amplitude of 35.6 V, and a conduction time of 200 msec and (**b**) at a 3.0-mm penetration depth, a signal amplitude of 32.6 V, and a conduction time of 300 msec. (**c**) RF-tissue reactions concentrated along the upper portion of the hair follicle with minimal to no RF-induced tissue coagulation in the hair bulb at a 3.0-mm penetration depth, a signal amplitude of 33.7 V, and a conduction time of 120 msec. (**d**) RF signals generate minimal tissue reaction on the upper portion of the hair follicle, with extensive RF tissue reaction around the hair bulb at a 2.0-mm penetration depth, a signal amplitude of 30.6 V, and a conduction time of 120 msec. H&E stain.

RF signals tended to propagate along the outside of hair follicles, mainly the outer root sheath and fibrous connective tissue, rather than through the inside of the hair follicle, which would have been the shortest path to the nearest electrode (Fig. 5a,b). Overall, hair follicular structures were mostly preserved with no histologic change. Basket-like vascular complexes surrounding the hair follicle were also notably congested. When RF tissue reactions were concentrated around the upper portion of the hair follicle (Fig. 5c), minimal to no RF-induced tissue coagulation was found in the hair bulb in the lower portion of the hair follicle. Meanwhile, when RF signals generated minimal tissue reaction throughout the upper portion of the hair follicle, more extensive RF tissue reactions were noted around the hair bulb (Fig. 5d).

## Discussion

RF signals can be emitted to produce electrothermal reactions in nerve fibers, muscles, skin, and tumors by bulk heating^1^,^2^,^3^,^4^,^8^. In skin, due to differences in the tissue impedances of skin layers, a water drop-shaped zone of electrothermal coagulation in the dermis is reportedly observed upon the delivery of RF energy to the skin through penetrating electrodes^4^,^9^,^10^. In this study, the histometric values for the width and depth of coagulation areas differed according to the depth of microneedle electrode penetration, despite treating with the same RF power and conduction time. In other words, deeper penetration with the microneedle electrodes generated larger columns of coagulation at the same RF power and conduction time. In addition to tissue impedances, the initiation and propagation patterns of RF energy can also contribute, in part, to the nature of RF-induced tissue coagulation^6^. In a previous study, the delivery of monopolar RF to *ex vivo* bovine liver tissue, which has relatively homogeneous tissue impedance and permittivity compared to the skin, induced RF tissue reactions along the entire length of the electrode, resulting in a maximal tissue response around the tip of the electrode^6^.

Bipolar RF devices equipped with insulated microneedle electrodes have been shown to generate electrothermal reactions at the tips of the electrodes similar to those for monopolar RF devices^7^. In the reported study, no remarkable RF-induced skin reactions were discovered in tissue between the electrodes at the conduction times of 20 msec, 50 msec, 100 msec, and 1000 msec and at signal amplitudes ranging from 5.0 V to 50.0 V^7^. However, such results are not consistent with those of other studies on invasive bipolar RF or the basic concepts of electrical conduction theory^1^,^2^,^3^,^4^,^5^. Theoretically, circuits of bipolar RF electrical current begin on an active anode and end on an active cathode, thereby generating RF tissue reactions along the nearest path between the two electrodes^2^. The results of this aforementioned study led us to question in what ways electrothermal reactions may differ with the conduction of bipolar RF energy through invasive non-insulated electrodes.

In the present study, we emitted bipolar RF energy through non-insulated penetrating electrodes at various conduction times to achieve serial RF tissue reactions on *in vivo* micropig skin, *ex vivo* micropig muscle, and *ex vivo* bovine liver tissues. In the *in vivo* micropig skin study, we found that invasive bipolar RF treatment using non-insulated penetrating electrodes also resulted in water drop- or cocoon-shaped oval coagulation columns, which were generated separately around each microneedle electrode in the dermis, at the conduction times of 120 msec, 200 msec, and 300 msec. While electrothermal changes along the epidermis were noticeable, the extents of injury were minimal in our experimental conditions, likely resulting from the epidermis’ tissue impedance and permittivity, as well as the initiation and propagation pattern of the bipolar RF signals. Additionally, all of the coagulation columns showed the same histologic findings, regardless of their location around the electrodes. Meanwhile, we found no evidence of the propagation of electrical current between the electrodes in the *in vivo* micropig skin study.

In the *ex vivo* bovine liver tissue study, we delivered bipolar RF energy for longer than 1 second to detect and analyse patterns in the formation and propagation of electrical currents therein. Due to more even impedance throughout the tissue, *ex vivo* bovine liver tissue rather than skin was chosen for study because the layers and appendage structures in the skin could significantly affect tissue impedance and permittivity. Compared to the *in vivo* micropig skin, *ex vivo* liver tissue, which is mainly composed of hepatocytes and vascular structures, required a longer conduction time to generate noticeable histologic changes. Nevertheless, the emission of RF energy for more than 1 second produced a cocoon-shaped area of coagulation around each microneedle electrode which is the area with higher current density, and then convergent areas of coagulation in the areas with lower current density between the microneedles as inter-electrode currents passed between electrodes: we suggest naming our novel finding of independent tissue coagulation around each electrode as a “Na effect” to distinguish it from other RF tissue reactions. In our experiments, the coagulation areas appeared sequentially with increased RF conduction time and could be easily induced at a low RF power. The first circuit of bipolar electrical current was formed between the pointed tips of the electrodes. Then, a second circuit appeared between the mid-portions of the electrodes. Finally, bipolar electrical current circuits were found along the entire length of the electrodes, as shown in the predictive model of invasive bipolar RF^5^. Additionally, after an extremely long conduction time, hepatocytes were realigned as hepatocyte filings in an electrical field by the bipolar electrical currents similar to those seen for iron filings under a magnetic field from a bar magnet.

In this study, noticeable changes were observed in the microvascular structures of the *in vivo* micropig skin and *ex vivo* bovine liver tissue. Electrical energy of alternating current seemed to be converted to heat near the outer layers of vascular structures and conducted through the walls of blood vessels^11^,^12^. Dermal microvasculature and perivascular structures between the electrodes seem to pass current better, show higher current density, and be selectively activated by bipolar RF current. RF-induced vascular tissue reactions were also found along the hair follicle, mainly the outer root sheath and fibrous connective tissues. Notwithstanding, the clinical significance of bipolar RF currents on blood vessels and hair follicles was not investigated in this study.

In conclusion, we found that the patterns of propagation of bipolar RF energy via non-insulated microneedle electrodes are similar to that with for monopolar RF energy using non-insulated electrodes. Moreover, by controlling RF conduction times, we showed that the delivery of bipolar RF energy via non-insulated electrodes can induce thermal responses similar to those for insulated electrodes. Although the histological compositions of micropig skin and muscle and bovine liver tissues do not exactly coincide with that of human skin, we believe that our histologic investigation into the electromagnetic initiation and propagation of RF tissue reactions induced by invasive bipolar non-insulated microneedle RF treatment will help guide further research and studies to advance RF technology for optimal treatment of various skin conditions.

## Methods

### Delivery of invasive bipolar RF via non-insulated microneedle electrodes

A bipolar 2-MHz RF device equipped with non-insulated microneedle electrodes (CELFIRM; Viol, Kyunggi, Korea) was utilized to evaluate RF tissue reactions on *in vivo* micropig skin and *ex vivo* micropig muscle and bovine liver tissue. When using this device, the treatment parameters of microneedle penetration depth, treatment mode, and power level were controlled to fit the study purpose. The device utilizes 10 mm × 10 mm disposable tips composed of 25 minimally invasive non-insulated microneedle electrodes uniformly arranged in a 5 × 5 pattern. The microneedles are constructed of 24 K gold-plated surgical stainless steel and comprise a body diameter of 300 ± 10 μm and a pointed microneedle tip length of 750 μm. The conductivity of stainless steel is 1.1 × 10^6^ S/m and that for gold as 4.1 × 10^7^ S/m.

### *In vivo* treatment of micropig skin with bipolar RF energy via invasive non-insulated electrodes

All experimental protocols were approved by the ethics committee of the Yonsei University Institutional Animal Care and Use Committee (2014–0150) and the methods are carried out in accordance with the approved guidelines. One female specific pathogen-free micropig (3-months old, weighing 8.4 kg) was used in all experiments. General anesthesia was administered via an intramuscular bolus injection of tiletamine/zolazepam (5 mg/kg) and xylazine (2 mg/kg). Thereafter, we performed endotracheal intubation and connected the pig to a ventilator. The lungs were ventilated with oxygen, and anesthesia was maintained with 2% isoflurane. Intravenous hydration with normal saline was maintained through a superficial auricular vein (25 ml/hour).

The back of the experimental micropig was shaved with an electrical razor, and the skin was subsequently marked with ink dots to outline 4-cm^2^ grids for each treatment parameter (a total of 216 grids); each grid was spaced at least 1 cm from the others to minimize RF and thermal effects on the other treatment areas. The operative field was cleansed with a mild soap and 70% alcohol. Then, the experimental micropig received minimally invasive bipolar microneedle RF treatment at electrode penetration depths ranging from 0.5 mm to 3.5 mm in increments of 0.5 mm; signal amplitudes ranging from 25.6 V to 36.6 V; and conduction times of 120 msec, 200 msec, and 300 msec. No cooling or topical anesthesia was applied before or after the treatment. The experimental micropig was sacrificed after sampling the treated skin in a humane manner according to standard protocols.

### *Ex vivo* treatment of bovine liver tissue with bipolar RF energy via invasive non-insulated electrodes

Prior to delivering invasive bipolar microneedle RF treatment on fresh bovine liver tissue, the surfaces thereof were marked with ink dots to outline 2-cm^2^ grids for each treatment parameter (a total of 37 grids); each grid was spaced at least 2 cm from the others to minimize RF and thermal effects on the other treatment areas. Treatment was performed at a penetration depth of 3.0 mm; a signal amplitude of 36.6 V; and conduction times of 120 msec, 200 msec, 300 msec, and 1–7 seconds in increments of 1 second. An additional experimental study was performed at a depth of 3.0 mm; signal amplitudes of 31.6 V and 36.6 V; and conduction times of 2, 3, and 4 seconds. Samples from the fresh bovine liver tissue were taken immediately after the treatment. All experiments were performed in triplet.

### Histological examination

At one hour after treatment, biopsy specimens of full thickness from the *in vivo* micropig skin study and *ex vivo* bovine liver tissue study were obtained for histologic evaluation. Each sample was fixed in 10% buffered formalin and then embedded in paraffin. Micropig skin and bovine liver tissue blocks were cut along the longitudinal plane to detect the insertion axes of the microneedle electrodes. For each treatment setting, 20 to 30 serial skin and liver tissue sections of 4-μm thickness were prepared and stained with hematoxylin and eosin. Additionally, 4-μm thick horizontal liver tissue sections were serially obtained and stained to evaluate inter-electrode interactions.

### High-speed cinematography

A high-speed digital video camera (Phantom v710; Vision Research Inc., Wayne, NJ, USA) set at a capture rate of 1,000 frames per second and a resolution of 1,280 × 800 pixels was utilized to record video footage of each individual tissue reaction, from initiation to vaporization of tissues, including the coalescence of coagulation areas and the oscillation of the resultant vaporizing bubble, over time.

Under two light-emitting diode spotlights (120 W), high-speed cinemagraphs captured the delivery of bipolar 2-MHz RF signals via non-insulated microneedle electrodes on *ex vivo* micropig muscle and bovine liver tissues. For optimized visualization, four linear non-insulated microneedle electrodes were inserted into the muscle and liver tissues to deliver RF signals with a maximum conduction time of 7 seconds and a signal amplitude of 50 V.

## Additional Information

**How to cite this article**: Na, J. *et al.* Electromagnetic Initiation and Propagation of Bipolar Radiofrequency Tissue Reactions via Invasive Non-Insulated Microneedle Electrodes. *Sci. Rep.* **5**, 16735; doi: 10.1038/srep16735 (2015).

## Supplementary Material

Supplementary Video 1a

Supplementary Video 1b

Supplementary Video Legends
