# Supplementary material for: Electromagnetic Initiation and Propagation of Bipolar Radiofrequency Tissue Reactions via Invasive Non-Insulated Microneedle Electrodes
Source: Sci Rep. 2015 Nov 13;5:16735. doi: 10.1038/srep16735 (PMC4643267; doi:10.1038/srep16735)
Supplement: Supplementary Video Legends [file srep16735-s3.pdf]

ARTICLE

Electromagnetic Initiation and Propagation of Bipolar Radiofrequency Tissue Reactions via Invasive Non-Insulated Microneedle Electrodes

Jongju Na[1], Zhenlong Zheng[2,3], Christopher Dannaker[4], Sang Eun Lee,[2] Jin-Soo Kang[5] & Sung Bin Cho[2,5]

[1]Department of Anatomy, Soonchunhyang University College of Medicine, Cheonan, Korea; [2]Department of Dermatology and Cutaneous Biology Research Institute, Yonsei University College of Medicine, Seoul, Korea; [3]Department of Dermatology, Yanbian University Hospital, Yanji, China; [4]Department of Dermatology, University of California, School of Medicine, San Francisco, CA, USA; [5]Kangskin Dermatology Clinic, Seoul, Korea.

Correspondence and requests for samples should be addressed to Sung Bin Cho, MD, PhD, Department of Dermatology and Cutaneous Biology Research Institute, Yonsei University College of Medicine, 50 Yonsei-ro, Seodaemun-gu, 120-752 Seoul, Korea. Tel.: +82.2.2228-2080. Fax: +82.2.313-9157. E-mail: sbcho@yuhs.ac

Total word count: 2,581
Number of references: 12
Number of tables: 0
Number of figures: 5
Number of supplementary videos: 1
Conflicts of interest: None declared
Funding sources: None

Running head: TISSUE REACTIONS WITH INVASIVE BIPOLAR RADIOFREQUENCY
Keywords: Electromagnetic signal, radiofrequency, microneedle, bipolar, invasive, micropig, bovine liver

SUPPLEMENTARY VIDEO LEGENDS

Video 1. High-speed cinematography of *ex vivo* liver tissue and micropig muscle treated with invasive bipolar RF using non-insulated microneedle electrodes. Tissue reactions using four linear non-insulated microneedle electrodes at a signal amplitude of 50V are initiated at the tips of the electrodes in *ex vivo* (**a**) liver tissue and (**b**) micropig muscle. Tissue reactions are then propagated upward along the entire length of the microneedle electrodes. Inter-electrode currents between neighboring electrodes become apparent first between the tips of the electrodes, second between the mid-portions of the electrodes, and lastly between the entirety of the electrodes.
